# NG2 proteoglycan as a pericyte target for anticancer therapy by tumor vessel infarction with retargeted tissue factor

**DOI:** 10.18632/oncotarget.6725

**Published:** 2016-01-05

**Authors:** Caroline Brand, Christoph Schliemann, Janine Ring, Torsten Kessler, Sebastian Bäumer, Linus Angenendt, Verena Mantke, Rebecca Ross, Heike Hintelmann, Tilmann Spieker, Eva Wardelmann, Rolf M. Mesters, Wolfgang E. Berdel, Christian Schwöppe

**Affiliations:** ^1^ Department of Medicine A, Hematology, Oncology and Pneumology, University of Muenster, Albert-Schweitzer-Campus 1, D-48129 Muenster, Germany; ^2^ Gerhard-Domagk Institute for Pathology, University of Muenster, Albert-Schweitzer-Campus 1, D-48129 Muenster, Germany

**Keywords:** truncated tissue factor, vascular targeting, vascular infarction, cancer, NG2 proteoglycan

## Abstract

tTF-TAA and tTF-LTL are fusion proteins consisting of the extracellular domain of tissue factor (TF) and the peptides TAASGVRSMH and LTLRWVGLMS, respectively. These peptides represent ligands of NG2, a surface proteoglycan expressed on angiogenic pericytes and some tumor cells. Here we have expressed the model compound tTF-NGR, tTF-TAA, and tTF-LTL with different lengths in the TF domain in *E. coli* and used these fusion proteins for functional studies in anticancer therapy. We aimed to retarget TF to tumor vessels leading to tumor vessel infarction with two barriers of selectivity, a) the leaky endothelial lining in tumor vessels with the target NG2 being expressed on pericytes on the abluminal side of the endothelial cell barrier and b) the preferential expression of NG2 on angiogenic vessels such as in tumors. Chromatography-purified tTF-TAA showed identical Factor X (FX)-activating procoagulatory activity as the model compound tTF-NGR with K_m_ values of approx. 0.15 nM in Michaelis-Menten kinetics. The procoagulatory activity of tTF-LTL varied with the chosen length of the TF part of the fusion protein. Flow cytometry revealed specific binding of tTF-TAA to NG2-expressing pericytes and tumor cells with low affinity and dissociation K_D_ in the high nM range. *In vivo* and *ex vivo* fluorescence imaging of tumor xenograft-carrying animals and of the explanted tumors showed reduction of tumor blood flow upon tTF-TAA application. Therapeutic experiments showed a reproducible antitumor activity of tTF-TAA against NG2-expressing A549-tumor xenografts, however, with a rather small therapeutic window (active/toxic dose in mg/kg body weight).

## INTRODUCTION

The formation of new blood vessels, supplying oxygen and nutrients as well as removing metabolic waste products, is essential for spread and metastasis of solid tumors [1]. Targeting tumor vasculature represents different therapeutic strategies, such as antiangiogenesis [2–4], vascular disruption [5, 6], and vascular targeting of antitumor molecules [7, 8].

**Figure 1:**
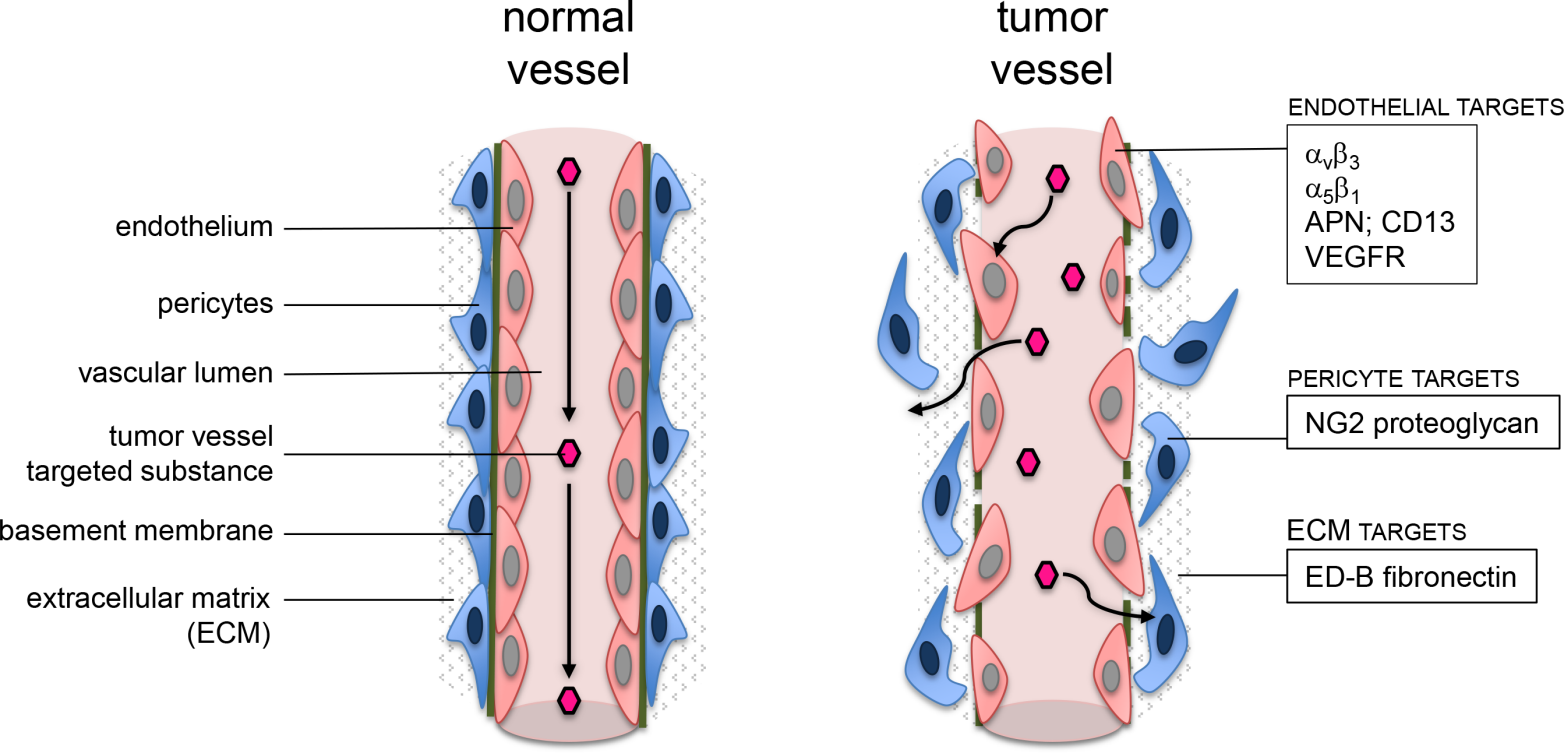
Schematic visualization of possible targets close to a tumor vessel wall

Direct contact to blood flow makes tumor vessel wall cells, such as endothelial cells and pericytes, easily accessible for antitumor agents. Denekamp et al. proposed tumor vessels and endothelial cells as a target for antitumor therapy [7]. Among various markers preferentially expressed in tumor vessel walls, NG2 (nerve/glial antigen 2) proteoglycan is of specific interest as a target [8]. NG2 is a transmembrane protein also known as high-molecular melanoma associated antigen [9–12]. It is expressed on some tumor cells of different histology [13, 14] and on pericytes of angiogenic in contrast to resting vessels such as in tumors [8, 9, 11, 15]. With this pattern, NG2 becomes a potential target for vascular delivery of antitumor drugs or mechanisms. Additionally, since tumor vessels - in contrast to normal vessels - show a leaky and permeable endothelial cell lining [16–19] which allows for extravasation of larger molecules into the tissue [20, 21] and because NG2 is expressed on pericytes covering the abluminal surface of endothelial cells, this could constitute a second barrier for selectivity leading to tumor vessel infarction induced by tissue factor (TF) retargeted to NG2 (Figure 1).

Use of coagulation factors to induce tumor vessel infarction was initiated by Thorpe et al. [22]. TF is a central initiator of the extrinsic coagulation pathway *in vivo*. Bound to the cell membrane it assembles a procoagulatory complex by binding Factor VIIa (FVIIa), and the TF:FVIIa complex initiates coagulation by cleaving FX to active FXa within a phospholipid milieu. The relative lack of coagulation-inducing activity of the soluble form of TF missing its transmembrane domain (truncated TF, tTF; [23]) can partly be reconstituted by relocalizing tTF into the proximity of the cellular phospholipid membrane [22]. Accordingly, targeting tTF via peptides and antibodies to different specific tumor vessel markers has led to rapid induction of thrombosis in tumor vessels [24–27].

Pasqualini et al. [28] revealed that small peptides containing the NGR motif (asparagine-glycine-arginine) bind to aminopeptidase N (APN; CD13). CD13 is a cell surface molecule with up-regulated expression on endothelial cells in tumors and tissues that undergo angiogenesis. We have constructed fusion proteins consisting of short NGR-peptide sequences coupled to the C-terminal end of tTF [29–31]. Several of these fusion proteins including tTF-NGR remain thrombogenic *in vitro*, bind to their respective targets on endothelial cells, and upon intravenous infusion induce thrombosis, vascular blood pooling and disruption in blood vessels in several human solid tumors growing in athymic mice with subsequent tumor growth retardation and regression. Intravenous infusion of tTF-NGR in cancer patients at dose levels without side effects was shown to reduce tumor blood flow *in situ* [30].

To improve selectivity of vascular infarction induced by these molecules we have chosen NG2 proteoglycan as an alternative target. Here we report for the first time retargeting of tTF by NG2-binding peptides TAASGVRSMH and LTLRWVGLMS selected by phage display and used for NG2 targeting before [8]. Additionally, all tTF-fusion proteins were constructed with different amino acid sequences and resulting molecular lengths of TF, since it was reported that shorter TF molecules show stronger procoagulatory efficacy [32]. With reference to this published observation we cloned full-length tTF_218_ or a variant which is 4 amino acids shorter as described by Magdolen et al. [32]. Chromatography-purified tTF-TAA showed identical FX-activating procoagulatory activity as the model compound tTF-NGR in Michaelis-Menten kinetics. The procoagulatory activity of tTF-LTL varied with the chosen length of the TF part of the fusion protein. Flow cytometry revealed specific binding of tTF-TAA to NG2-expressing pericytes and tumor cells with low affinity. *In vivo* and *ex vivo* fluorescence imaging of tumor xenograft-carrying mice and of the explanted tumors revealed a reduction of tumor blood flow upon tTF-TAA application. Therapeutic experiments showed a reproducible antitumor activity of tTF-TAA against NG2-expressing A549-tumor xenografts, however, with a rather small therapeutic window (active/toxic dose in mg/kg body weight (bw)).

## RESULTS

### Expression and detection of NG2 proteoglycan

**Figure 2:**
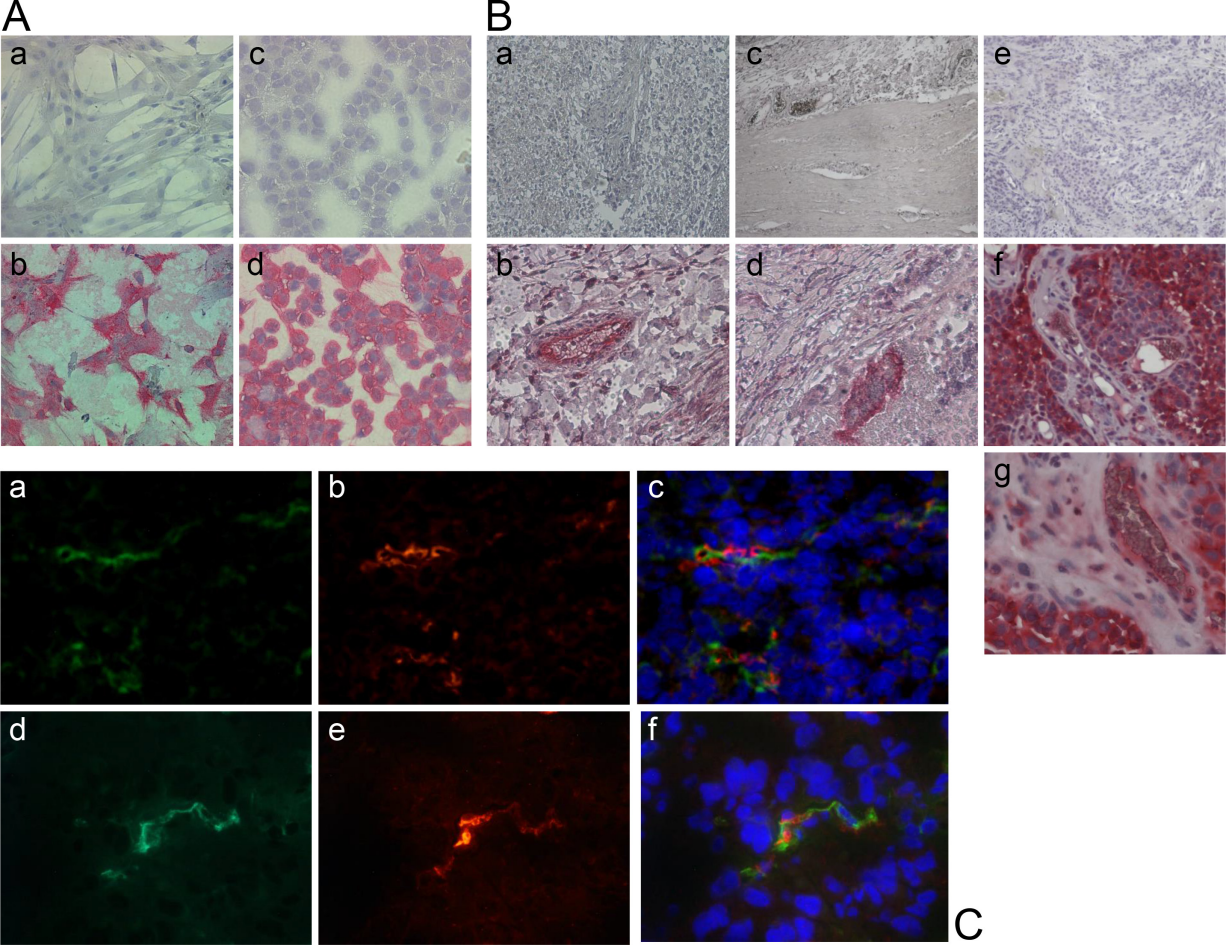
Expression of the proteoglycan NG2 in human cell lines and tumor tissue (**A**) APAAP staining with an anti-NG2 antibody shows NG2 expression on the surface of human aortic smooth muscle cells (HuAoSMC, b) and human G361-melanoma cells (d). Cells only incubated with the secondary antibody were used as controls (a, c). (**B**) The APAAP staining of different resected human tumor tissues reveals the expression of the proteoglycan NG2 especially outside of the tumor endothelium in non-small cell lung carcinoma (NSCLC, b), colon carcinoma (d) and melanoma (g), and on the surface of melanoma cells (f). Tissue sections only incubated with the secondary antibody were used as controls (a, c, e). (**C**) Human tumor xenotransplants in mice (HT1080 fibrosarcoma, upper row; U87 glioblastoma, lower row) were resected and immunostained for NG2 (red; b, e) and for CD31 (green; a, d), respectively. Co-staining with both antibodies proves the co-localization of NG2-expressing pericytes and CD31-expressing endothelial cells (c, f). Nuclei were stained with DAPI (blue).

In a first series of experiments we tested for expression of the chosen target NG2 in human aortic smooth muscle cells (HuAoSMC; representing pericytes) and some human tumor cell lines of different histology using immunohistology. Figure 2A summarizes examples showing strong protein expression of NG2 in both, HuAoSMC and G361-melanoma cells. Next we investigated NG2 expression in human tumor tissue sections of different histology. Immunohistology showed strong expression of NG2 in the tumor vessel walls of e.g. non-small cell lung cancers (NSCLC) and colorectal carcinomas without expression by the tumor cells (Figure 2B). In contrast, we have also detected tumors expressing NG2 not only on the vessel walls, but also by the tumor cells (for a human metastasized melanoma, see Figure 2B, f and g). To validate our *in vivo* xenograft models used for experimental therapy, we have performed fluorescence immunohistology on explanted human tumor xenografts to detect NG2 and endothelial cell vessel marker CD31 (PECAM-1). Figure 2C depicts results for a human fibrosarcoma and a human glioblastoma as examples, showing CD31-positive endothelial cell tubes with neighboring NG2 expression representing vessel coating pericytes.

### Cloning, expression and purification of tTF-fusion proteins

**Figure 3:**
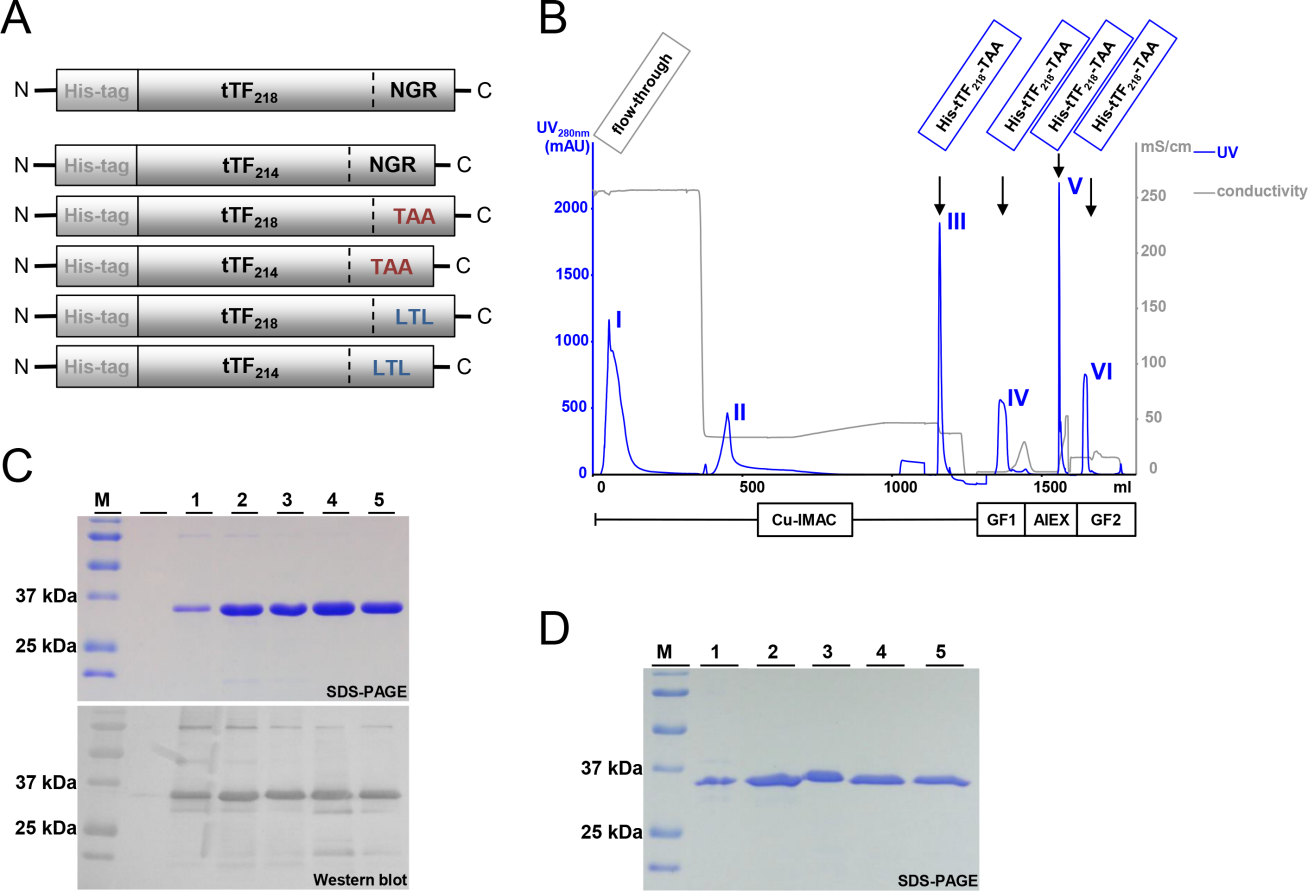
Schematic illustration of the NG2-targeting tTF-fusion protein constructs and their purification process (**A**) The N-terminal His-tag allows the purification of tTF constructs by metalchelate affinity chromatography; the tTF domain consisting of 214 or 218 amino acids, respectively, mediates the coagulation activity; the C-terminal peptide binding motif enables the binding to the tumor endothelium (CD13 via NGR binding motif in the model compound tTF-NGR) or to the tumor pericytes (NG2 via TAA or LTL binding motif), respectively. (**B**) The four-step purification process of the tTF-fusion proteins comprises an immobilized metal affinity chromatography (IMAC), a gel filtration/buffer exchange step (GF1), an anion exchange chromatography (AIEX), and a final gel filtration/buffer exchange step (GF2). The elution peaks of the particular purification steps are exemplarily shown for tTF_218_-TAA. (**C**) As an example, purified tTF_218_-TAA fusion protein (molecular weight: ∼30 kDa) and the purification intermediates analyzed by SDS-PAGE and Western blotting with an anti-tTF antibody are shown: (M) molecular weight standard; (1) IMAC flow-through; (2) IMAC eluate; (3) GF1 eluate; (4) AIEX eluate; (5) end product (GF2 eluate). (**D**) SDS-PAGE analysis of the tTF_214_-LTL purification course. For abbreviations, see above.

tTF-NGR as a model fusion protein with two different tTF lenghts, tTF_214_-NGR and tTF_218_-NGR, tTF_214_-TAA and tTF_218_-TAA with the NG2-binding C-terminal peptide TAASGVRSMH [8], as well as tTF_214_-LTL and tTF_218_-LTL with the C-terminal NG2-binding peptide LTLRWVGLMS [8] (for structure see Figure 3A) were cloned into the pET30a(+) expression vector and expressed in *E. coli* as described in the Materials and Methods section. Subsequently, tTF-fusion proteins were purified and refolded by a 4-step chromatography-based procedure as described [31] and yielded uniform peaks as shown for tTF_218_-TAA as an example in Figure 3B. SDS-PAGE (Figure 3C and 3D) and Western blotting with anti-TF antibodies (Figure 3C) proved high purity of the products. The final yield of the purified tTF-fusion proteins was comparable to that of the model compound tTF_218_-NGR with the exception of tTF_218_-LTL, which yielded considerable lower amounts.

### Activation of Factor X (FX) by the different tTF-fusion proteins

**Figure 4:**
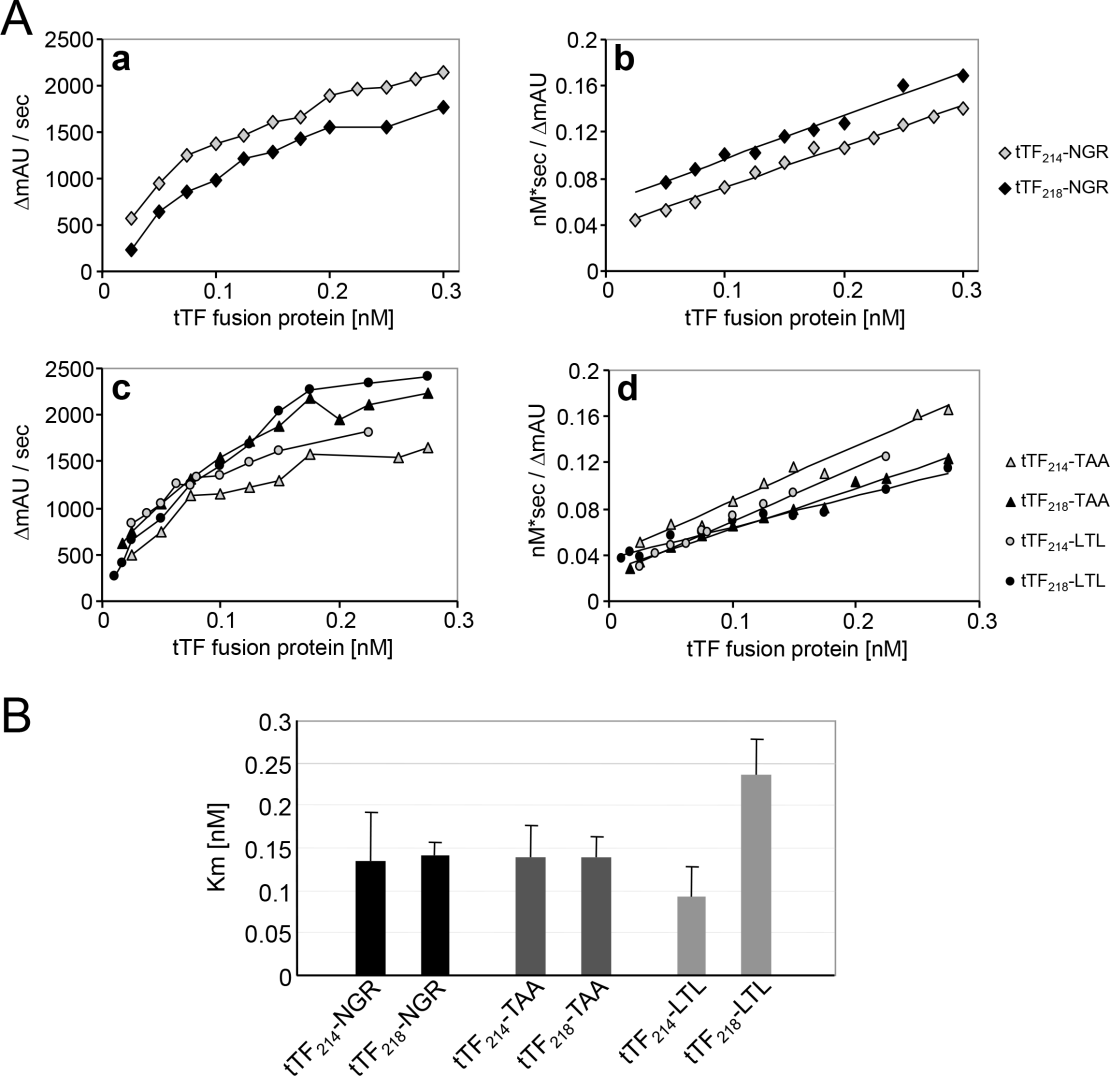
Factor X activation assay of the different tTF proteins (**A**) The ability of the fusion proteins to enhance the specific proteolytic activation of FX by FVIIa in the presence of phospholipids was evaluated by Michaelis-Menten analysis (a, c). The Michaelis constants (K_m_) of the activation were calculated by hyperbolic regression analysis according to Hanes et al. [33] (b, d). (**B**) The determined K_m_ values of all tTF constructs are summarized in this scheme. There are no significant differences between the respective short and long constructs as analyzed by two-sided *t*-test: tTF-NGR: *p* = 0.85; tTF-TAA: *p* = 0.97; tTF-LTL: *p* = 0.056. Data are presented as means +/− standard errors.

Next, we assayed the activation of FX induced by all 5 tTF-fusion proteins and compared it to the model compound tTF_218_-NGR (Figure 4). Michaelis constants (K_m_) calculated by hyperbolic regression analysis [33] for the different tTF-fusion proteins within the TF:Factor VIIa complex were between 0.093 and 0.253 nM (Figure 4), which is comparable with the K_m_ values established for tTF_218_-NGR before [29–31]. However, in contrast to the results published by Magdolen [32], the shorter tTF_214_-fusion proteins did not reveal a stronger procoagulatory activity (Figure 4B). On the other hand, the long variant of tTF-LTL showed a considerably higher K_m_ representing a lower procoagulatory activity when compared to all other fusion proteins. This led us to the selection of candidate compounds for the following experiments.

### Cellular binding studies by flow cytometry

**Figure 5:**
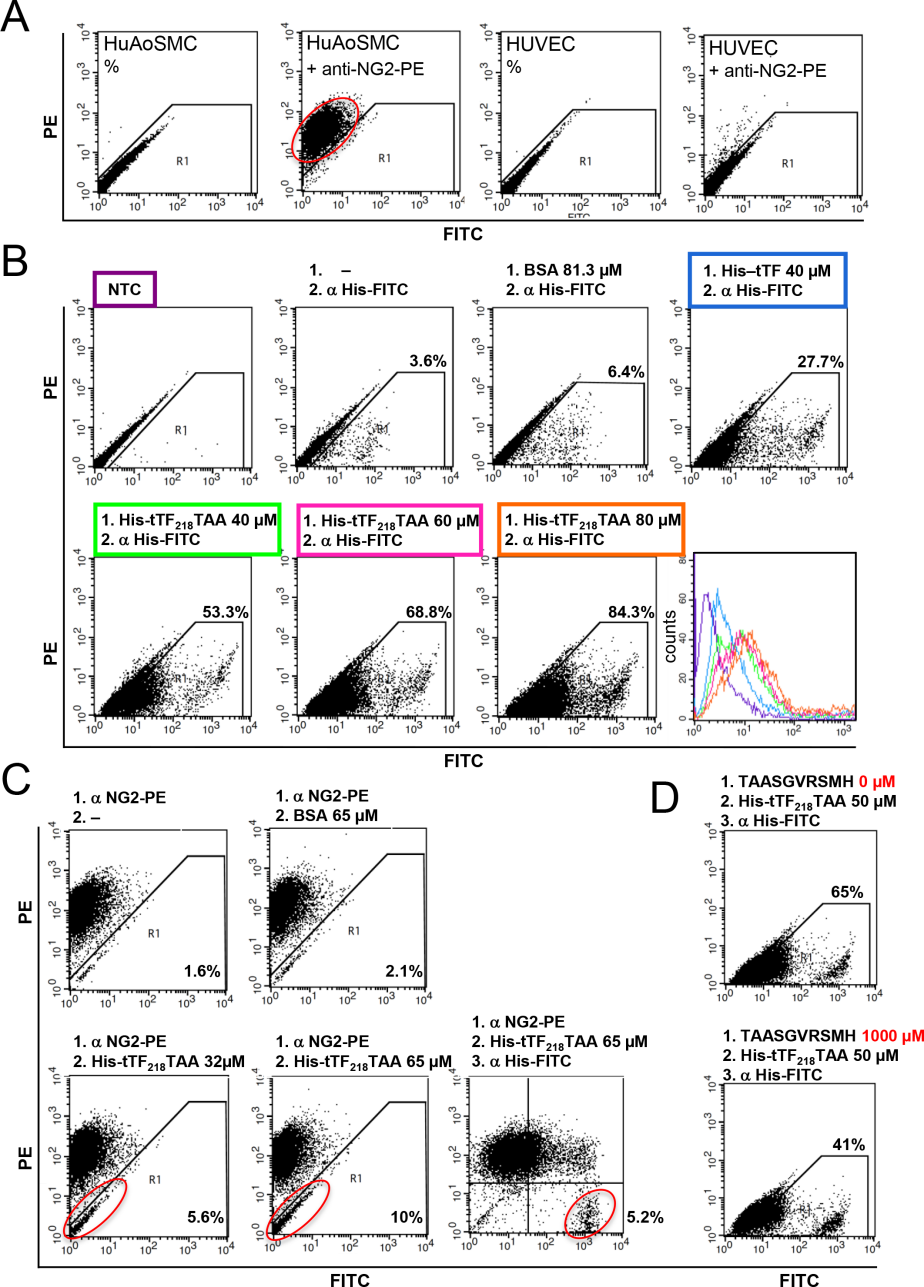
Binding of tTF_218_-TAA to human aortic smooth muscle cells (HuAoSMC) as measured by flow cytometry (**A**) The presence of NG2 on HuAoSMCs and HUVECs was detected with a monoclonal PE-labeled anti-NG2 antibody. (**B**) Dose-dependent binding of tTF-TAA to NG2-expressing HuAoSMCs (lower row): cells were incubated with different concentrations of tTF-TAA. Bound fusion protein was then detected with a FITC-labeled anti-His antibody (α His-FITC). tTF without targeting TAA-peptide binds unspecifically to the cells, but in a clearly smaller amount when compared to tTF-TAA (upper row, right panel). Untreated cells (NTC), cells only incubated with the anti-His antibody, or cells incubated both with the control protein BSA and the anti-His antibody, respectively, where used as controls (upper row). A summary of all binding curves is shown in the histogram (lower row, right panel); for color assignment see boxes above the respective panels. (**C**) Blocking of HuAoSMCs using the PE-labeled anti-NG2 antibody (α NG2-PE) and displacement by tTF-TAA: after incubation with the α NG2-PE antibody, cells were incubated with different concentrations of tTF-TAA. The fusion protein was able to displace some of the bound antibody in a dose-dependent manner (lower left and middle panels). Bound tTF-TAA fusion protein was then detected with the anti-His-FITC antibody (lower right panel). Displacement of the NG2-PE antibody by the control protein BSA was not effective (upper middle panel). (**D**) In a further setup, tTF-TAA binding was blocked by pre-incubation with 20-fold excess of pure TAASGVRSMH-decapeptide (lower panel). Bound tTF-TAA fusion protein was then detected with the anti-His-FITC antibody. Cells without pre-incubation were used as controls (upper panel).

We have characterized target-specific binding of tTF_218_-NGR on activated endothelial cells before [30]. In the next set of experiments we have used different NG2 proteoglycan-expressing cells for binding studies with tTF-TAA. Figure 5 summarizes essential results for NG2-specific binding of tTF_218_-TAA to human aortic smooth muscle cells (HuAoSMC), representing activated pericytes. NG2 was expressed on HuAoSMC in contrast to human umbilical vein endothelial cells (HUVEC) (Figure 5A) and we detected concentration/dose-dependent binding of His-tagged-tTF-TAA to HuAoSMC by His-tag specific secondary antibody (Figure 5B). NG2 specificity of the binding was proven since tTF-TAA could dose-dependently replace a NG2-specific antibody from its binding sites (Figure 5C) and preincubation of the cells with excess amounts of a small TAASGVRSMH-decapeptide inhibited tTF-TAA binding dose-dependently (Figure 5D).

**Figure 6:**
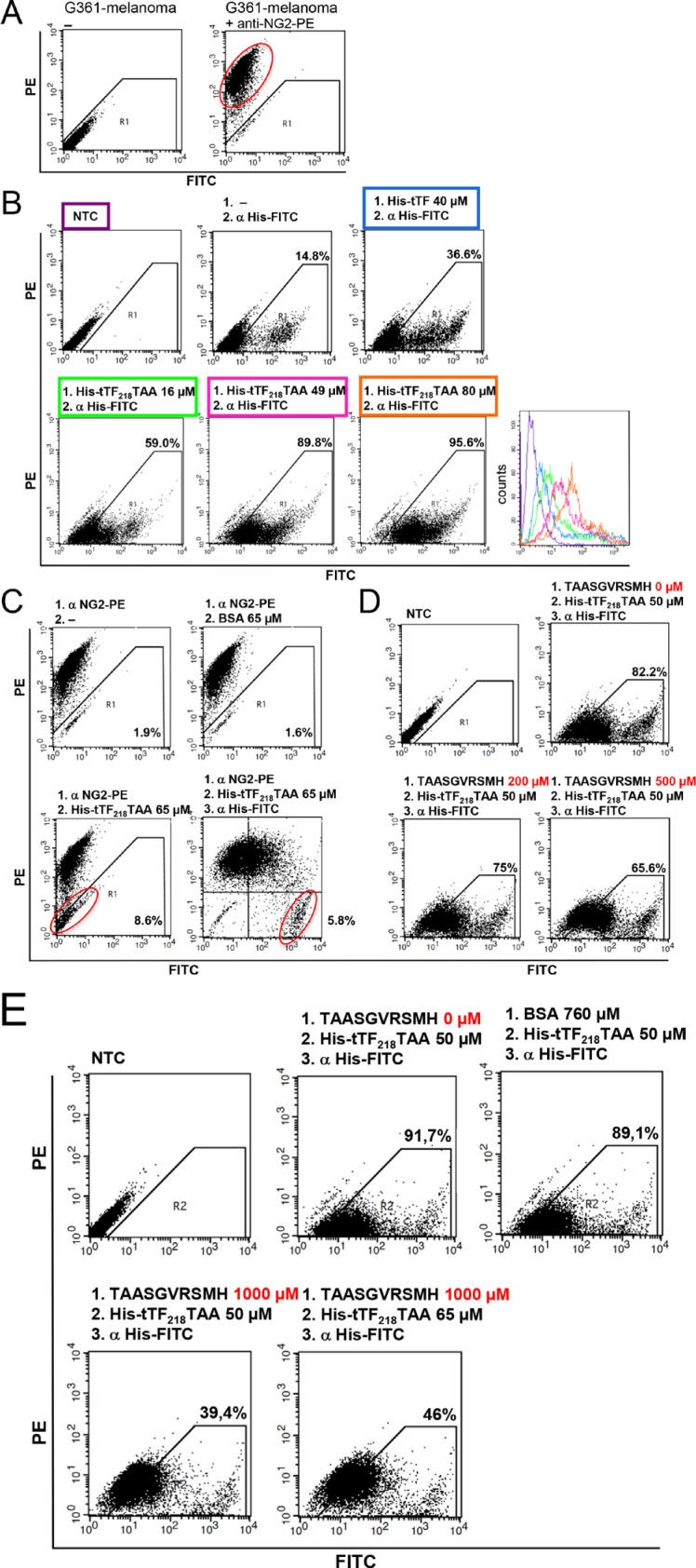
Binding of tTF_218_-TAA to human G361-melanoma cells as measured by flow cytometry (**A**) The presence of NG2 on G361-melanoma cells was detected with a monoclonal PE-labeled anti-NG2 antibody. (**B**) Dose-dependent binding of tTF-TAA to NG2-expressing G361 cells (lower row): cells were incubated with different concentrations of tTF-TAA. Bound fusion protein was then detected with a FITC-labeled anti-His antibody (α His-FITC). tTF without targeting TAA-peptide binds unspecifically to the cells, but in a clearly smaller amount when compared to tTF-TAA (upper row, right panel). Untreated cells (NTC) or cells only incubated with the anti-His antibody, respectively, where used as controls (upper row, left and middle panel). A summary of all binding curves can be seen in the histogram (lower row, right panel); for color assignment, see boxes above the respective panels. (**C**) Blocking of G361 with the PE-labeled anti-NG2 antibody (α NG2-PE) and displacement by tTF-TAA: after incubation with α NG2-PE antibody cells were incubated with tTF-TAA, whereupon the fusion protein was able to displace some of the bound antibody (lower left panel). Bound tTF-TAA fusion protein was then detected with the α His-FITC antibody (lower right panel). Displacement of the α NG2-PE antibody by the control protein BSA was not effective (upper right panel). (**D, E**) In a further setup, tTF-TAA binding was blocked by pre-incubation with 4- to 20-fold excess of pure TAASGVRSMH-dekapeptide in a dose-dependent manner (D, E lower panel). Bound tTF-TAA fusion protein was then detected with the α His-FITC antibody. Untreated cells and cells without pre-incubation were used as controls (upper panel). By increasing the applied concentrations of fusion protein, more already bound TAASGVRSMH could be displaced (E, lower right panel). Blocking of tTF-TAA binding by the control protein BSA was not effective (E, upper right panel).

Comparable experimental results were obtained using the NG2-expressing melanoma cell line G361 (Figure 6).

### Binding assays with ^123−^I-tTF_218_-TAA

**Figure 7:**
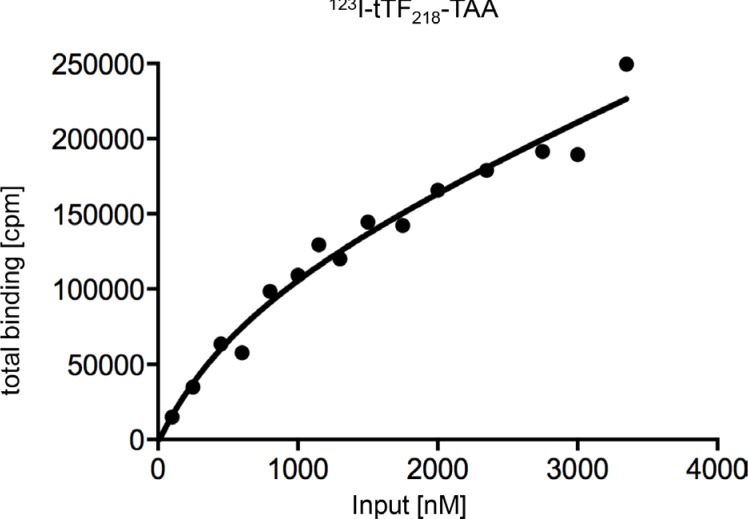
Determination of the dissociation constant (K_D_) of tTF_218_-TAA binding to NG2-expressing HuAoSMC to characterize the binding affinity Binding assays with different concentrations of radiolabeled tTF-TAA (^123^I-tTF-TAA) and cultivated human aortic smooth muscle cells (HuAoSMC) were performed and the amount of bound fusion protein was carried out in a Berthold gamma counter. Determination of K_D_ was performed with a nonlinear curve fitting program (GraphPad Prism 6: one site – total binding) and analysis of total-binding data by means of the equation for (one-site) total binding revealed a K_D_ of 785 nM. This equation (total binding = {(Bmax * [L]/(K_D_+[L])} + NS * [L]) assumes that unspecific binding is commensurate to the concentration of the radioligand and that only a small amount of the radioligand binds, so that the input-concentration is virtually identical with the concentration of free radioligand (for further details see Materials and Methods).

To further characterize binding of the candidate fusion protein tTF-TAA to NG2 on HuAoSMC, the protein was iodinated following standard procedures and incubated with HuAoSMC at increasing concentrations, in some competition assays with up to 15-fold excess of non-iodinated tTF-TAA protein. We obtained some binding saturation at high input concentrations of approx. 3 μM (Figure 7). In addition to classical Scatchard Analysis [34], application of a non-linear curve-fitting program revealed non-linear kinetics. With due caution, data indicated the possibility of more than 1 binding site and a K_D_ at approx. 785 nM. This is, as expected, characteristic for low-affinity binding of the material. However, these data only represent an approximation of the actual binding behaviour of tTF-TAA.

### *In vivo* and *ex vivo* imaging of tTF_218_-TAA in xenograft-bearing mice and explanted tumors

**Figure 8:**
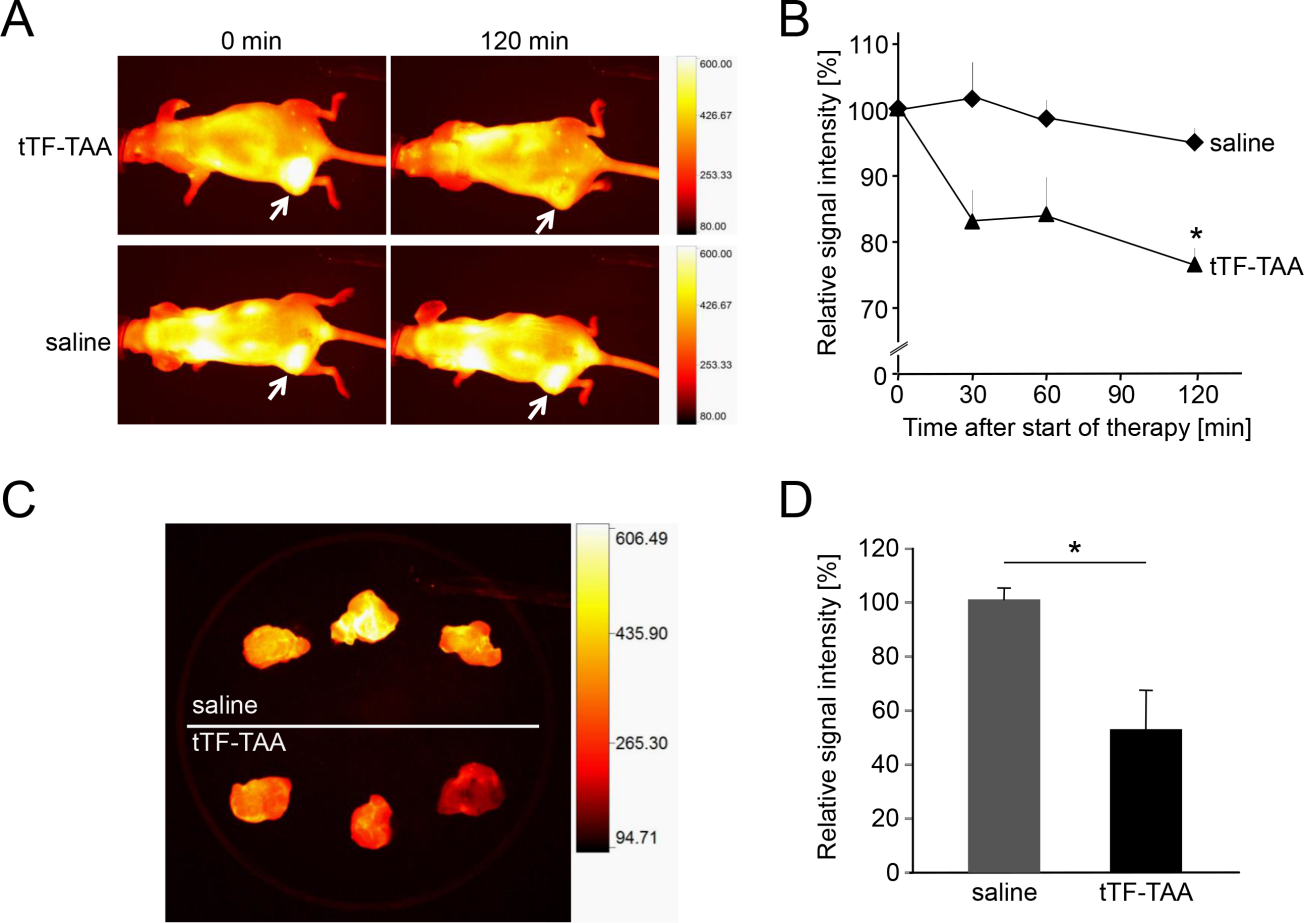
Treatment with NG2-targeting tTF_218_-TAA *in vivo* and *ex vivo* as monitored by fluorescence reflectance imaging (FRI) (**A, B**) FRI was performed with A549-bearing CD-1 nude mice that received AngioSense^®^ as an *in vivo* blood pool- and tumor-imaging agent 22 h prior to the start of the therapy. AngioSense^®^ signal intensities in tumors were measured at time point zero (0 min; see white arrows) and set as 100%. After the application of 1 mg/kg tTF_218_-TAA or saline, respectively (each with *n* = 3), signal intensities were analyzed for 120 min and revealed a significant decrease of the signal in treated tumors when compared to the saline controls (B; asterisk denotes statistical significance, *p* < 0.05). Data are presented as means +/− standard errors. (**C, D**) AngioSense^®^ fluorescence intensities of explanted tumors showed an even more significant difference between treated tumors (C, lower row) and saline controls (C, upper row), which was quantified as shown in D (asterisk denotes statistical significance, *p* < 0.05). Data are presented as means +/− standard errors.

To visualize and quantify the reduction of tumor blood flow by systemic application of tTF-TAA, we have used athymic CD-1 mice bearing the human A549 NSCLC xenografts for fluorescence reflectance imaging (FRI) with the *in vivo* blood pool- and tumor-imaging agent AngioSense^®^. Intravenous tTF-TAA reduced tumor blood flow significantly already after 30 min reaching a maximum at 120 min (Figure 8A and 8B). At this time the tumor vascular fluorescence by AngioSense^680®^ was significantly blocked in comparison to saline controls. *Ex vivo* analysis of the xenografts revealed identical effects, however, being even better visible (Figure 8C and 8D).

### Effect of tTF_218_-TAA on growth of human tumor xenotransplants

**Figure 9:**
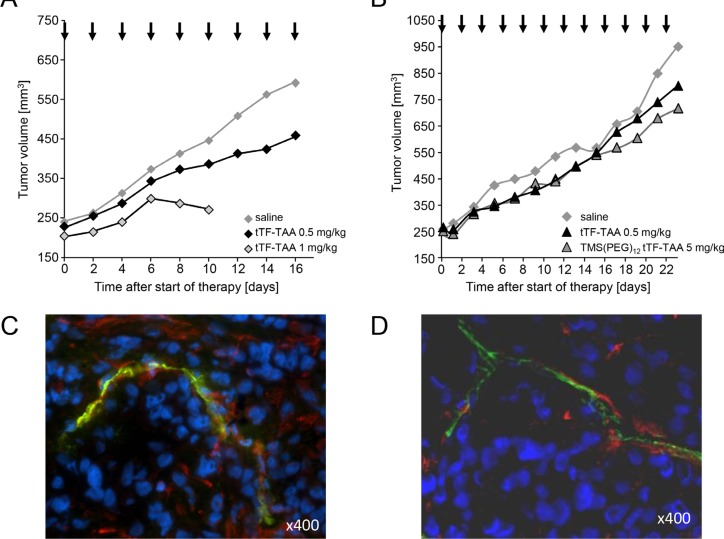
Effect of tTF_218_-TAA on the growth of human xenotransplants in CD-1 nude mice (**A**) Tumor growth retardation of human adenocarcinoma of the lung (A549) xenotransplanted into athymic CD-1 mice after i.v. administration of 0.5 (*n* = 5) and 1 mg/kg bw (*n* = 4) tTF_218_-TAA versus 0.9% saline control (*n* = 8). Therapy started at an average tumor size of 240 mm^3^ and was continued for 16 days on every second day; arrows indicate time points of injection. Due to side effects, the 1 mg/kg therapy cohort had to be stopped at day 10. (**B**) Effect of 0.5 mg/kg BW tTF_218_-TAA (*n* = 8), 5 mg/kg bw TMS(PEG)_12_ tTF-TAA (*n* = 9) and 0.9% saline control (*n* = 9), respectively, on the growth of human M21 melanoma xenotransplanted into CD-1 nude mice. Therapy started at an average tumor size of 250 mm^3^ and was continued for 23 days on every second day; arrows indicate time points of i.v. administration. The experiment had to be terminated with separating growth curves on day 23 due to tumor size and animal regulations. (**C, D**) Vessel decoration with NG2 of explanted control tumors. Human tumor xenotransplants in mice (×400; C: A549 adenocarcinoma of the lung; D: M21 melanoma) were resected and co-immunostained for NG2 (red) and for CD31 (green), respectively. Nuclei were stained with DAPI (blue).

Subsequently, the *in vivo* antitumor activity of tTF_218_-TAA was determined in athymic CD-1 nude mice bearing different human xenografts (A549 NSCLC and M21 melanoma, respectively; Figure 9). Drugs and controls (saline) were slowly injected i.v. at the doses and time intervals as shown in Figure 9. Repeated injections of 0.5 and 1 mg tTF_218_-TAA/kg bw induced tumor growth delay for a longer period of time when compared with saline controls in A549 adenocarcinoma of the lung (Figure 9A). Toxicity observed up to 0.5 mg/kg bw was some tail tip necrosis upon repeated injections and some cutaneous hematomas. However, there was a rather small therapeutic window (active/toxic dose in mg/kg bw) since repeated application of 1 mg/kg bw led to toxicity and the consecutive stop of the experiment at this dose level due to paralysis of the hind legs and sudden death. A second independent experiment (details not shown) with A549 tumor-bearing mice revealed a similar tumor-growth retarding effect of 0.5 mg/kg BW tTF_218_-TAA (*n* = 8) as compared to saline (*n* = 8). Tumor size on day 16 reached borderline significance for values expressed as percentage (*p* = 0.088, two-sided *t*-test) comparing the 0.5 mg/kg bw groups of both experiments with the saline controls. Experiments with M21 melanoma xenografts (Figure 9B) showed a trend for tumor growth retardation by tTF_218_-TAA either in the native or in a randomly TMS(PEG)_12_-PEGylated form. However, this did not reach statistical significance, since experiments had to be stopped due to tumor size and animal regulation. The vessel decoration of the explanted xenograft tumors at the end of these experiments showed parallel staining of vessel structures with CD31 and NG2 for the A549 tumors (Figure 9C), as well as for the M21 tumors (Figure 9D). Additionally, the A549 tumor cells clearly showed expression of NG2, in M21 tumor cells NG2 expression was less visible (Figure 9C and 9D).

In contrast, colon tumor xenografts showing either a low expression of the target NG2 on the tumor vessels (KM12L4; data not shown), or higher expression, however without clear colocalization to CD31+ endothelial cells indicating blood flow-allowing microvessels (HTB38; data not shown), did not respond with tumor growth retardation to systemic tTF_218_-TAA (data not shown). Non-responsiveness of these colon xenografts were also found upon treatment with higher doses of randomly TMS(PEG)_12_-PEGylated tTF_218_-TAA (data not shown). Random PEGylation was found to allow higher doses of tTF fusion proteins before [35].

## DISCUSSION

Peptide-targeted tTF possibly have some advantages over antibodies or larger antibody fragments as targeting moieties for tTF. Larger antibody molecules show low tumor penetration [36], nonspecific accumulation and uptake in the reticulo-histiocytic system (RHS), and immunogenicity. Our tTF-fusion proteins consist of small peptides coupled to the C-terminus of tTF [29–31, 35]. By connecting the peptide to the C-terminus of tTF, the tTF moiety is part of the tTF:FVIIa complex adopting a physiological orientation perpendicular to the phospholipid membrane [37], which leads to a potent initiation of coagulation. Thrombogenic activity of i.v. application of the model compound tTF-NGR in tumor vasculature with subsequent tumor vessel infarction and growth delay could be shown in several tumor mouse models. First-in-man experiences with low doses of tTF-NGR (1–4 mg/m^2^ by i.v. infusion) showed inhibition of tumor perfusion without any side effects as “proof of principle” [30].

While tTF-NGR is entering early clinical trials, we have launched a search for improving pharmacokinetics [35] and alternative targets. With respect to the latter, we here report our attempts to improve selectivity of vascular infarction induced by these molecules by choosing NG2 proteoglycan as an alternative target. NG2 is expressed on some tumor cells of different histology [13, 14] and on pericytes of angiogenic in contrast to resting vessels such as in tumors [8, 9, 11, 15] and thus becomes a potential target for vascular delivery of antitumor proteins. Possibly conflicting with our targeting approach is the expression pattern of NG2 in several normal tissues as shown on the protein level (http://www.proteinatlas.org/ENSG00000173546-CSPG4/tissue). However, this does not necessarily provide accessibility of NG2 to proteins transported by the blood stream, and for the mechanism of action of proteins such as tTF-TAA a coagulation-competent milieu is a prerequisite. Since tumor vessels - in contrast to normal vessels - show a leaky and permeable endothelial cell lining [16–19] which allows for extravasation of larger molecules into the tissue [20, 21] and because NG2 is expressed on pericytes on the abluminal surface of endothelial cells, we hypothesized the presence of a second barrier for selectivity leading to angiogenic tumor vessel infarction induced by tTF-TAA.

We report for the first time retargeting of truncated tissue factor by NG2-binding peptides TAASGVRSMH and LTLRWVGLMS [8] and retardation of tumor growth by tumor vessel infarction. The fusion proteins tTF-TAA and tTF-LTL were obtained with two different lengths of the tTF-moiety. In contrast to what was reported earlier [32], the different molecular length of the tTF proteins did not improve procoagulatory activity, rather the full-length tTF_218_-TAA showed identical FX-activating potential when compared to the model compound tTF-NGR. Furthermore, flow cytometry revealed specific binding of tTF-TAA to NG2-expressing pericytes and NG2-expressing tumor cells with low affinity. *In vivo* and *ex vivo* fluorescence imaging of tumor xenograft-bearing animals and the explanted tumors showed vascular occlusion and reduction of tumor blood flow upon tTF-TAA application. Therapeutic xenograft experiments showed a reproducible antitumor activity of tTF-TAA against NG2-expressing A549-tumor xenografts.

However, the therapeutic activity of NG2-targeting tTF-TAA, when compared to tTF-NGR in identical xenograft models targeting CD13 on endothelial cells [30, 31, 35], was considerably lower, and the therapeutic window of tTF-TAA upon repeated application (active/toxic dose in mg/kg bw) was rather small. Reasons for this observation are speculative. Since NG2 is located on the outward side of the vessel wall, the leakiness of the endothelial barrier might not be sufficient to allow concentrations of tTF-TAA on target high enough to induce coagulation and tumor vessel infarction. Low expression of the target on pericytes, variable and low pericyte coverage on tumor vessels, or suboptimal phospholipid milieu for procoagulatory activity might be other limiting factors. In consequence, trapping of the molecule inside the tumor may be low and systemic concentrations of the fusion protein after multiple injections might reach procoagulatory levels elsewhere. Limiting toxicity of tTF-NGR with a therapeutic window of 1:5 (therapeutic cose:LD_10_) in the mouse is pulmonary embolism [35].

In conclusion, tTF-TAA was selected as a new candidate fusion protein retargeting tissue factor to the pericyte target NG2, a surface proteoglycan expressed on angiogenic vessels and some tumor cells. The protein retained similar FX-activating procoagulatory activity as the model compound tTF-NGR, it revealed specific binding to the NG2 target on pericytes and tumor cells, and showed a reproducible antitumor activity against NG2-expressing A549-tumor xenografts. However, due to the small number of tumors characterized as being sensitive as xenografts towards tTF-TAA and its rather small therapeutic window, this NG2-targeted fusion protein shows no improved therapeutic profile over the lead compound tTF-NGR.

## MATERIALS AND METHODS

### Cloning strategies, expression and HPLC purification of the tTF-fusion proteins

Cloning strategy of tTF-TAA was based on the expression vector pET-30a(+) (Novagen, Schwalbach am Taunus, Germany), already containing an N-terminal histidine tag (His-tag) for subsequent purification of the protein, and the cDNA sequence encoding for the tTF-NGR fusion protein (amino acids 1-218 of TF and the C-terminal heptapeptide GNGRAHA (His-tag-tTF_1-218_-GNGRAHA); pET30a(+)/tTF-NGR), as described earlier [29–31]. By site-directed mutagenesis, one of the two *Tat* I restriction sites was changed into an *Kpn* I site, leading to an expression vector in which the sequence of TF amino acids 203 to 218 and the C-terminal fused NGR-heptapetide is flanked by sole *Tat*I and *BamH*I restriction sites (pET30a(+)^2KpnI^/tTF-NGR). An 84 bp fragment containing the sequence of the tTF amino acids 203–218 and the sequence of the decapeptide TAASGVRSMH was synthesized by Eurofins MWG Operon (Ebersberg, Germany) and subcloned in a pCR2.1Topo vector. By digestion with the enzymes *Tat*I and *BamH*I this 84-bp fragment was isolated and then ligated into the expression vector pET30a(+)^2KpnI^/tTF-NGR pretreated with the same restriction enzymes. The generated tTF-TAA construct possesses the TF amino acids 1-218 with the C-terminal fused TAA-decapeptide and an N-terminal histidine tag for subsequent purification of the protein by using immobilized metal affinity chromatography (IMAC).

The vector pET30a(+)^2KpnI^/tTF_218_-TAA was introduced into competent *Escherichia coli* cells (BL21 DE3) according to the manufacturer's protocol (Novagen). Protein expression and purification has been described in detail earlier [29–31, 35]. Briefly, after stimulation with IPTG (Novagen), the cells were harvested and lyzed with 5–7 ml of a lysozyme-containing lysis buffer, incubated for 90 min at room temperature (RT) and centrifuged at 12.000g for 20 min and 4°C. The pellet was resuspended and homogenized by sonicating in washing buffer (10 mM Tris-HCl, pH 7.5, 1 mM ethylenediaminetetraacetic acid (EDTA), 3% Triton X-100). The inclusion bodies were solubilized with guanidinium buffer by an overnight incubation at room temperature. After centrifugation (12.000g for 30 min) the supernatant was filtered through a 0.22-μm filter and stored at −25°C. Further purification and refolding were carried out by a multistep high-performance liquid chromatography (HPLC)-based purification process with an ÄKTA purifier system (GE healthcare, München, Germany). The first capture step consists of an IMAC with copper (Chelating Sepharose 6FF, GE healthcare) using copper as chelating agent. The N-terminal histidine-tagged tTF-TAA fusion protein binds to the immobilized copper ions so that the complete refolding (urea gradient from 6 to 0 M) and washing processes are performed on the column, from which the protein is eluted by applying 300 mM imidazole. During the subsequent gel filtration, the IMAC eluate is conditioned by a buffer-exchanging step using Sephadex G-25 (GE healthcare) in order to prepare for the following intermediate purification step. This anion-exchange chromatography step (AIEX, Q Sepharose HP, GE healthcare) allows further separation of the eluted proteins according to differences in their net charges. Moreover, it removes most of the remaining impurities such as other proteins, nucleic acids, and endotoxin. The concluding polishing step again comprises a gel filtration using Sephadex G-25 in order to remove remaining trace impurities and to exchange the buffer to phosphate-buffered saline (PBS). The final protein solution is stored at −25°C.

The other tTF-fusion proteins as shown in Figure 2 were produced and purified along the lines described for tTF_218_-TAA above.

### TMS(PEG)_12_ PEGylation

Random TMS(PEG)_12_ PEGylation of the tTF_218_-TAA protein was performed according to the manufacturer's protocol (Thermo Scientific/Pierce, Bonn, Germany; see also Schwöppe et al. [35]). Briefly, the protein (solved in PBS) was incubated for 2 h at 4°C with an 30-fold excess of TMS(PEG)_12_, a trimethyl succinimidyl polyethylene glycol ester (molecular weight: 2420.8 Da), which reacts with primary amino groups (such as lysine) of the tTF-TAA protein, releasing NHS. To remove the NHS group and excess TMS(PEG)_12_, the reaction mixture was purified by a HPLC-based gel filtration with Sephadex G-25 medium (GE Healthcare, München, Germany). The final PEGylated protein solution is stored at −25°C.

### Factor X activation by tTF fusion proteins

We have assayed the activity of our retargeted TF-fusion proteins to act as a cofactor in the membrane-bound TF:FVIIa complex activating FX to FXa by protease function and visualized this reaction by a Spectrozyme Xa assay as described by Ruf et al. [38]. The ability of the varying concentrations of the tTF fusion proteins with constant concentrations of the other components of the procoagulatory assembly of TF:FVIIa, FX and phospholipids to enhance the specific proteolytic activation of FX by FVIIa was assessed by Michaelis-Menten analysis as described by Ruf et al. [38]. Briefly, 20 μl of the following was added to each well in a microtiter plate: (a) 50 nM recombinant FVIIa (Novo-Nordisc, Bagsværd, Denmark) in TBS containing 0.1% bovine serum albumin (BSA); (b) 0.1–1.5 nM of e.g. tTF-TAA protein in TBS-BSA; (c) 25 mM CaCl_2_ and 500 μM phospholipids (phosphatidylcholine/phosphatidylserine, 70/30, MM; Sigma, München, Germany). After 10 min at room temperature, the substrate FX (Enzyme Research Laboratories, Swansea, UK) was added (final concentration 1 μM). After additional 10 min, the reaction was stopped in 100 mM EDTA and Spectrozyme FXa (American Diagnostica, Greenwich, USA; final concentration 0.7 mM) was added. The rates of FXa generation were monitored by the development of color at 405 nm with a microplate reader (Bio-Rad, München, Germany). The Michaelis constant (K_m_) of the FX activation induced by varying concentrations of the tTF-fusion protein within the complex with FVIIa was calculated by hyperbolic regression analysis according to Hanes [33].

### Cell cultures

Human aortic smooth muscle cells were purchased from PromoCell (Heidelberg, Germany) in passage 2 and only used at low passage numbers. Cells were cultured in DMEM/F12 medium supplemented with 10% fetal calf serum (FCS) and 2.5 mM glutamine (Gibco, Eggenstein, Germany) and maintained at 37°C in 5% CO_2_ and high humidity. Cell culture dishes were coated with 0.2% gelatine. The human G361-melanoma cell line was cultured in McCoy 5A medium supplemented with 10% FCS and 1.5 mM glutamine. Cells, which both grow adherent and in suspension, were obtained from ATCC (Manassas, VA, USA). The human A549-lung carcinoma cell line was purchased from ATCC and cultured in Ham's F12 medium supplemented with 10% FCS and 1 mM glutamine. The human malignant melanoma cell line M21 was kindly provided by Dr. Siletti (University of California, San Diego, CA, USA) and cultured in RPMI 1640 medium supplemented with 10% FCS and 2 mM glutamine. M21 cells grow both adherent and in suspension. The colon carcinoma cell lines HTB-38 and KM12L4 were cultured in IMDM medium supplemented with 10% FCS. HTB-38 cells were purchased from ATCC; KM12L4 cells were kindly provided by Dr. I.J. Fidler (The University of Texas M.D. Anderson Cancer Center, Houston, TX, USA). The human glioblastoma cell line U87-MG (ATCC) was cultured in MEM medium supplemented with 10% FCS, 1 mM pyruvate (Gibco) and 1% non-essential amino acids (Gibco). Tumor cell lines were subjected to short tandem repeat (STR) profiling for identity confirmation. For some lines (e.g. M21) we established own STR profiles.

### *In vitro*-binding studies using flow cytometry

NG2 expression of human cell lines and primary cells, and differential binding of His-tagged tTF-fusion proteins to NG2-expressing cells was analyzed by flow cytometry using the BD FACS Calibur flow cytometer (Becton-Dickinson (BD), San Jose, CA, USA). Briefly, 90% confluent cells were trypsinized (10% Trypsin, Gibco), washed twice with PBS and blocked with human immunoglobulin G (IgG, 1 μg/1 × 10^5^ cells). For direct staining of the cell surface protein NG2, cells were incubated with the monoclonal mouse anti-NG2-phycoerythrin (PE) antibody (R & D-Systems, Minneapolis, MN, USA; 0.25 μg/1 × 10^5^ cells) for 30-45 minutes at 4°C. After two washing steps with ice-cold PBS/10% FCS, cells were resuspended in 500 μl PBS/FCS and analyzed in the flow cytometer. For specific binding analysis, IgG-blocked cells were incubated with 16–80 μM tTF-fusion proteins (or tTF as control) for 24 min at 37°C. After incubation and one washing step with ice-cold PBS/10% FCS, the monoclonal fluoresceinisothiocyanate (FITC)-labeled mouse-anti-histidine-tag antibody was used for detection of bound protein. Briefly, cells were incubated with 0.5 μg mouse anti-6xHis-FITC antibody per 1 × 10^5^ cells for 30 min at 4°C, washed twice with PBS/FCS, resuspended in 500 μl PBS/FCS and analyzed in the flow cytometer. To show competition for binding sites, IgG-blocked cells were pre-incubated/blocked with 0.125 μg mouse anti-NG2-PE antibody for 45 min at 4°C in the dark, so that nearly all cells were PE-positive. After washing with PBS/FCS, binding studies were performed as described above. In a second setup, cells were pre-incubated/blocked with excess amounts of e.g. pure TAA-decapeptide (TAASGVRSMH, 200–1000 μM = 4–20-fold excess) for 16 min at 37°C; the tTF fusion protein-binding studies were performed as described above.

### *In vitro*-binding studies using iodinated tTF-TAA

To further characterize binding of the candidate fusion protein tTF-TAA to NG2 on HuAoSMC, the protein was iodinated following standard procedures and incubated with HuAoSMC at increasing concentrations, and in some competition assays with up to 15-fold excess of non-iodinated tTF-TAA protein. Production capacity for tTF-TAA limited this second competition assay, as we could not use the 100–1000-fold excess of cold material as usually done for classical Scatchard Analysis [34]. Instead, we additionally used a non-linear curve-fitting program (GraphPad Prism 6: *one site - total binding*) where it is possible to determine the K_D_ directly by fitting the total binding without the determination of unspecific binding as an approximation.

### Tumor xenograft models

All procedures on animals were performed in agreement with German regulations (Tierversuchsgesetz §8 Abs. 2) and specifically approved in form of a project license. CD-1 nude mice were purchased from Charles River Laboratories (Sulzfeld, Germany) and acclimated to our animal-experiment facility for at least 1 week before any experimentation. Mice were maintained in individually-ventilated cages (IVC) on a 12:12 h light:dark cycle in a low-stress environment (22°C, 50% humidity, low noise) and given food and water *ad libitum*.

Single cell suspensions of the different tumor cells were injected subcutaneously (s.c.) into the right anterior flank of female CD-1 nude mice (9–12 weeks old). For therapeutic experiments, tumor growth was allowed to a mean volume of approx. 200–400 mm^3^. Mice were randomly assigned to different experimental groups. The tTF_218_-TAA fusion protein (0.5 or 1 mg/kg bw, respectively) was slowly applied intravenously (i.v.) via the tail veins. Animals in control groups received 0.9% NaCl solution. Injections were repeated every second day for nine times (first therapy approach) or for 11 times (second therapy approach). Tumor size was evaluated using a standard caliper measuring tumor length and width; tumor volumes were calculated using the standardized formula (length × width^2^> × π/6). According to our project license, animals had to be sacrificed when tumors became too large, if mice lost > 20% of bw, or at signs of pain. In this case, mice were sacrificed by cervical dislocation in deep ketamine/xylazine anesthesia in agreement with standard regulations and the project license.

### Histology

For *histological analysis of primary cells or cell cultures*, respectively, cells were seeded on gelatin-coated culture slides (BD Bioscience, San Jose, CA, USA; 8 chambers, 1 × 10^4^ cells/well) and cultured for 36 hours. After three washing steps with PBS, the chamber was removed and cells were fixated on slides by incubation with methanol for 15 min. Finally, cells were washed with Tris-buffered saline (TBS, 3 × 5 min) and used for APAAP staining (alkaline phosphatase and anti-alkaline phosphatase method, modified from Cordell et al. [39], see below). Histological analyses of human tumor tissues were performed with formalin-fixed and paraffin-embedded tissues. After deparaffinization according to standard protocols, tumor slides were washed with both aqua dest. and TBS, and used for APAAP staining. To this end, sections were incubated with the primary antibody rabbit-anti-NG2 (Merck Millipore, Schwalbach, Germany; dilution 1:100) for 16 hours at 4°C in a humid chamber. After washing with TBS, slides were incubated with the first bridge antibody (mouse anti-rabbit-IgG, Dako, Hamburg, Germany; dilution 1:100) followed by the incubation with the second bridge antibody (rabbit anti-mouse-IgG, Dako; dilution 1:25) each for 30 min at RT in a humid chamber. Finally, after three TBS-washing steps, samples were incubated with the APAAP complex (Dako; dilution 1:50; 30 min) and after further TBS-washing steps with the substrate solution consisting of substrate buffer with *Fast Red* and Levamisol (Dako; approx. 30 min). After adequate staining, reaction was stopped with aqua dest., followed by counterstaining the nuclei with Mayer's hemalum solution for 8 sec.

*Histological analyses of xenograft tumor tissues* were performed with O.C.T.-embedded and cryo-conserved tissues according to standard protocols. Briefly, tissues were embedded in Tissue-TEK O.C.T. (Sakura, Alphen aan den Rijn, The Netherlands), snap-frozen in liquid nitrogen, and stored at −85°C. Frozen samples were cut to 5-μm sections and transferred onto glass slides. H & E-staining was performed according to standard procedures.

For *immunofluorescence staining*, slides were fixed with acetone/methanol, and dried according to standard protocols. Sections were incubated with an anti-NG2 antibody (see above; dilution 1:200) followed by a tetramethylrhodamine (TRITC)-labelled goat anti-rabbit-IgG antibody (red; Dianova, Hamburg, Germany; dilution 1:100). Co-staining was performed with a rat anti-mouse-CD31 antibody (BD Bioscience; dilution 1:20) followed by a fluorescein isothiocyanate (FITC)-labelled goat anti-rat-IgG antibody (green; Dianova; dilution 1:100). Nuclei were stained using 4′, 6-diamidino-2-phenylindole (DAPI).

### Fluorescence reflectance imaging (FRI)

CD-1 nude mice bearing A549 xenotransplants received 2 nmol (150 μl, i.v.) of the fluorescent *in vivo* blood pool- and tumor-imaging agent Angiosense^®^ 680 Ex (PerkinElmer, Rodgau, Germany). After 22 hours, the tumor accumulation of the fluorescent contrast agent was detected and quantified with the *in vivo* imaging system FX PRO (Bruker BioSpin MRI GmbH, Ettlingen, Germany) using a 630 nm-excitation filter and a 700 nm-emission filter (time point t_0_). After treatment with a single dose of 1 mg/kg tTF_218_-TAA or 0.9% saline, respectively, *in vivo* tumor fluorescence intensities were analysed and quantified over a 120 minutes period. During FRI studies, animals were anesthetized by isoflurane inhalation (2–3%, together with 1–1.5 l/min O_2_ ventilation). Finally, mice were sacrificed and signal intensities of explanted tumors were quantified.

### Statistical analyses

Statistical significances of differences between the different groups were tested by the *t* test or by Mann-Whitney rank sum test for independent groups. Two-tailed *P* values lower than 0.05 were considered as indicating significant differences.

## Abbreviations

AIEX: anion exchange chromatography
APAAP: alkaline phosphatase-anti-alkaline phosphatase
APN: aminopeptidase N
BSA: bovine serum albumin
bw: body weight
DAPI: 4′,6-diamidino-2-phenylindole
DTT: dithiothreitol
EDTA: ethylenediaminetetraacetic acid
FRI: fluorescence reflectance imaging
FCS: fetal calf serum
FITC: fluorescein isothiocyanate
FVIIa: factor VIIa
FX: Factor X
GF: gel filtration
HPLC: high performance liquid chromatography
HuAoSMC: human aortic smooth muscle cells
IMAC: immobilized metal ion affinity chromatography
IVC: individually-ventilated cage
LTL: LTLRWVGLMS
LD_10_: lethal dose for 10% of animals treated upon one i.v. injection (within 24 h)
NG2: nerve/glial antigen 2
NGR: peptide GNGRAHA
NHS: N-hydroxysuccinimid
NSCLC: non-small cell lung cancer
PECAM-1: platelet endothelial cell adhesion molecule 1
PBS: phosphate-buffered saline
PE: phycoerythrin
PEG: polyethylene glycol
RHS: reticulo-histiocytic system
RT: room temperature
SDS-PAGE: sodium dodecyl sulphate polyacrylamide gel electrophoresis
TAA: peptide TAASGVRSMH
TBS: Tris-based saline
TF: tissue factor
tTF: truncated tissue factor
TMS(PEG)_12_: trimethyl succinimidyl polyethylene glycol ester
TRITC: tetramethylrhodamine isothiocyanate
